# Maternal septicemia caused by *Streptococcus mitis*: a possible link between intra-amniotic infection and periodontitis. Case report and literature review

**DOI:** 10.1186/s12879-022-07530-z

**Published:** 2022-06-20

**Authors:** Piya Chaemsaithong, Waranyu Lertrut, Threebhorn Kamlungkuea, Pitak Santanirand, Arunee Singsaneh, Adithep Jaovisidha, Sasikarn Pakdeeto, Paninee Mongkolsuk, Pisut Pongchaikul

**Affiliations:** 1 Department of Obstetrics and Gynecology, Faculty of Medicine, Ramathibodi Hospital, Mahidol University, 270 Rama VI Rd. Ratchathewi, Bangkok, 10400 Thailand; 2 Department of Pathology, Faculty of Medicine, Ramathibodi Hospital, Mahidol University, 270 Rama VI Rd. Ratchathewi, Bangkok, 10400 Thailand; 3 Chakri Naruebodindra Medical Institute, Faculty of Medicine, Ramathibodi Hospital, Mahidol University, 111 Bang Pla, Bang Phli, Samut Prakan, 10540 Thailand; 4 Integrative Computational BioScience Center (ICBS), Mahidol University, Nakhon Pathom, Thailand; 5 Institute of Infection, Veterinary and Ecological Sciences, University of Liverpool, Liverpool, UK

**Keywords:** Chorioamnionitis, Dental caries, Intra-amniotic infection, Microbial invasion of amniotic cavity, Periodontal disease, Periodontitis, Preterm, Preterm PROM, Septicemia

## Abstract

**Background:**

Intra-amniotic infection has a strong causal association with spontaneous preterm birth and preterm prelabor rupture of membranes (PPROM). The most common route of intra-amniotic infection is the ascending pathway in which microorganisms from the vagina gain access to the amniotic cavity. Distant microorganisms such as those from the oral cavity have been reported in intra-amniotic infection through hematogenous spreading.

**Case presentation:**

A 31-year-old gravida 1, para 0 Thai woman at 33^+6^ weeks’ gestation presented with leakage of vaginal fluid and irregular uterine contraction. She developed fever at 4 h after admission and was later diagnosed with acute chorioamnionitis. A Cesarean section was performed to terminate pregnancy. In addition to a blood culture, the cultures of amniotic fluid, vaginal and chorioamniotic membrane swabs were positive for *Streptococcus mitis* with identical susceptibility profiles. After the delivery and antibiotic prescription, oral examination showed dental caries and chronic periodontitis.

**Conclusions:**

This is the first case report demonstrating maternal septicemia and intra-amniotic infection caused by *S. mitis* which might be attributed to periodontitis in women presenting with preterm PROM. We highlighted the association of periodontal disease and preterm labor/PROM syndrome. Oral cavity examination should be included in the prenatal care to ensure good dental hygiene.

## Background

Intra-amniotic infection and/or inflammation is causally linked with spontaneous preterm birth and preterm prelabor rupture of membranes (PPROM) [1, 2]. The frequencies of intra-amniotic infection/inflammation in preterm labor and PPROM are approximately 30% and 50%, respectively [1–7]. While an ascending migration from the vagina is preponderant, pathogens may also gain access to the amniotic cavity by other pathways such as hematogenous dissemination or accidental introduction at the time of an invasive prenatal procedure [1, 2, 8–14]. Bearfield et al. identified similar strains of *Fusobacterium nucleotum (F. nucleotum)* and *Streptococcus* spp. from dental plaque and amniotic fluid, which suggests an oral origin of amniotic fluid microorganism infection [15]. Subsequently, other oral bacteria such as *Capnocytophaga *spp., *Rothia dentocariosa* and *Eikenella corodens* have also been detected in amniotic fluid [15–24].

*Streptococcus mitis (S. mitis)*, a member of viridans streptococci, is prevalent in the normal flora in the oropharynx [25, 26]. There have thus far been two case reports of acute histological chorioamnionitis caused by *S. mitis* [27, 28]. Both studies showed that *S. mitis* was identified in placental culture. Antecedent cunnilingus and dental scaling are proposed to be associated with *S. mitis* infection [28]. One report described that *S. mitis* is one of the polymicrobial microorganisms detected in the mid-trimester amniotic fluid of women who subsequently had fetal death [29].

Herein, we reported a unique case of *S. mitis* septicemia in a woman presenting with PPROM and clinical chorioamnionitis. Samples of amniotic fluid, vagina, chorioamniotic membranes, and maternal blood were taken for aerobic cultures. The results demonstrated similar strains of *S. mitis*. The patient was subsequently confirmed to have deep dental caries and chronic periodontitis. This is the first case report demonstrating septicemia, clinical chorioamnionitis and intra-amniotic infection may be presumably due to chronic periodontitis.

## Case presentation

A 31-year-old gravida 1, para 0 Thai woman at 33 weeks and 6 days of gestation presented at the labor and delivery unit due to the leakage of amniotic fluid at 26 h prior to the admission. Her antenatal care was unremarkable except for morbid obesity (body mass index 44.5 kg/m^2^) and pre-gestational diabetes. She was administered 81 mg of aspirin and 16 units of insulin daily. Her fasting and two-hour postprandial blood sugar ranged between 106–123 and 99–131 mg/dL, respectively. Her blood test, serology and ultrasound abnormality examination were normal. She had no underlying disease and denied a history of smoking, alcoholic drinking, or illicit drug use.

At 33^+6^ weeks’ gestation, she complained of vaginal leaking fluid and irregular uterine contraction 26 h prior to the admission. She had no fever or vaginal bleeding. Her fetal movement was good. Her vital signs showed no abnormalities. A dry sterile speculum examination revealed clear pooling fluid in the vagina with positive cough and nitrazine tests. Vaginal examination showed the cervix was 2 cm dilated with the effacement of 25%. Her white blood cell count (WBC) was 13,450 cells/mm^3^ with 80% neutrophil. Urinalysis was normal. External fetal monitoring demonstrated normal fetal heart rate and variability. Transabdominal ultrasonography examination demonstrated that the fetal growth was normal; however, the amniotic fluid index was only 2 cm. Therefore, we did not perform any amniocentesis for the determination of intra-amniotic infection/inflammation status. Expectant management was performed; steroid as well as antibiotic therapy (intravenous ampicillin, azithromycin) were given to promote lung maturity and prolong latency period, respectively.

The patient developed fever (38.2 °C) around 4 h after admission. Her pulse and blood pressure were 90 bpm and 121/71 mmHg, respectively. Continuous cardiotocography demonstrated a fetal heart rate of 165 bpm with moderate variability. Uterine contractions were 3-min intervals with moderate intensity. No uterine tenderness or foul-smelling discharge was found. Her WBC and C-reactive protein (CRP) were elevated (WBC 17,940 cells/mm^3^ with 85.4% neutrophil, CRP 24.36 mg/dL). Clinical chorioamnionitis was diagnosed and oxytocin was given to augment labor progression. The antibiotics were changed to intravenous ceftriaxone (2 g/every 24 h), intravenous metronidazole (500 mg/every 8 h), and oral clarithromycin (500 mg/every 8 h) to treat intra-amniotic infection as recommended in previous studies [30–33]. Her cervical progression was arrested at 4 cm for 4 h; hence, Cesarean delivery was performed. She delivered a male fetus with Apgar score of 8, 10 at 1 min and 5 min after delivery, respectively. Birth weight of the baby was 2680 g.

**Fig. 1:**
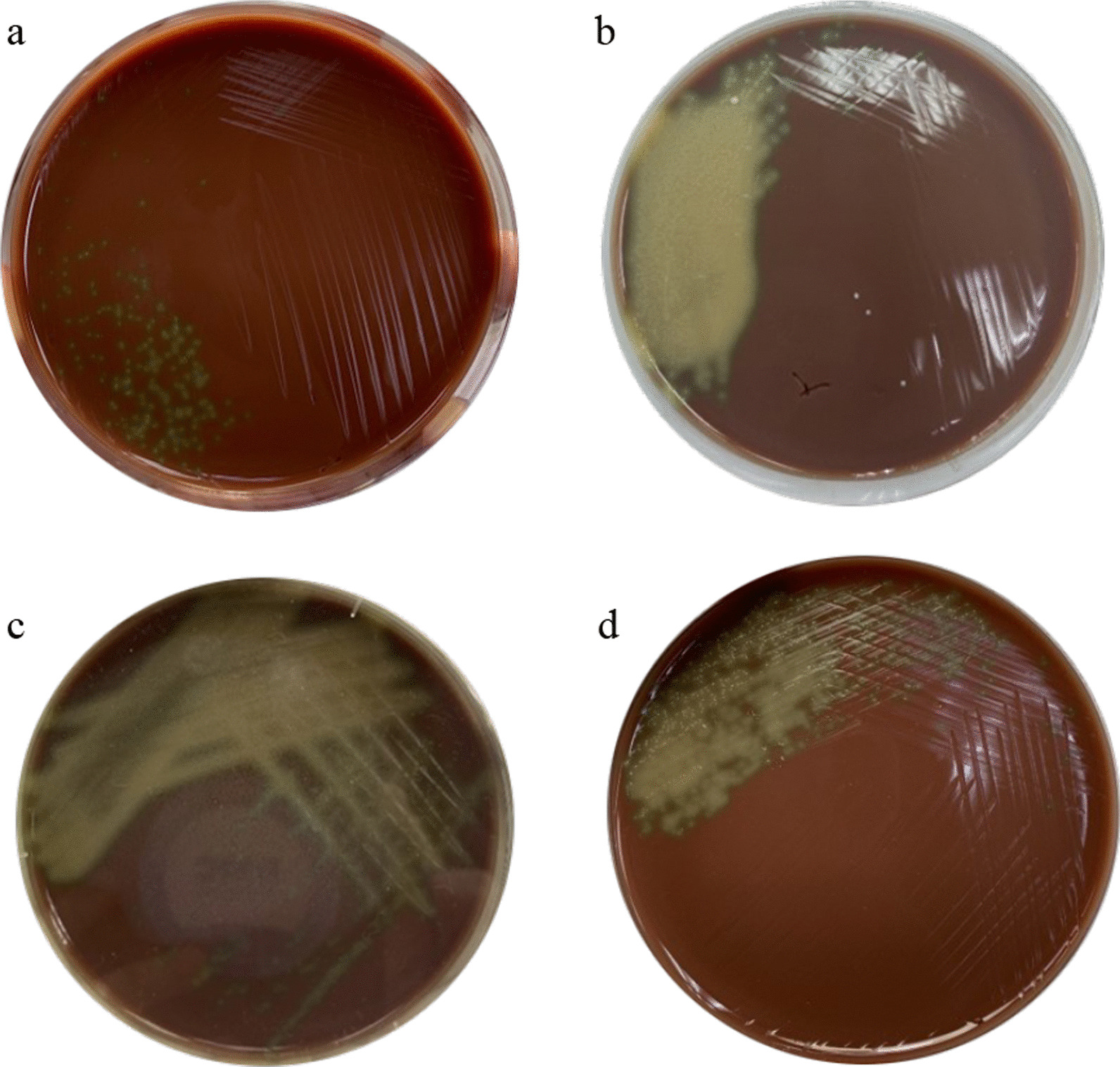
Blood agar plate showing colonies obtained from: **a** peripheral blood; **b** vaginal swab; **c** amniotic fluid and; **d** placenta

**Fig. 2:**
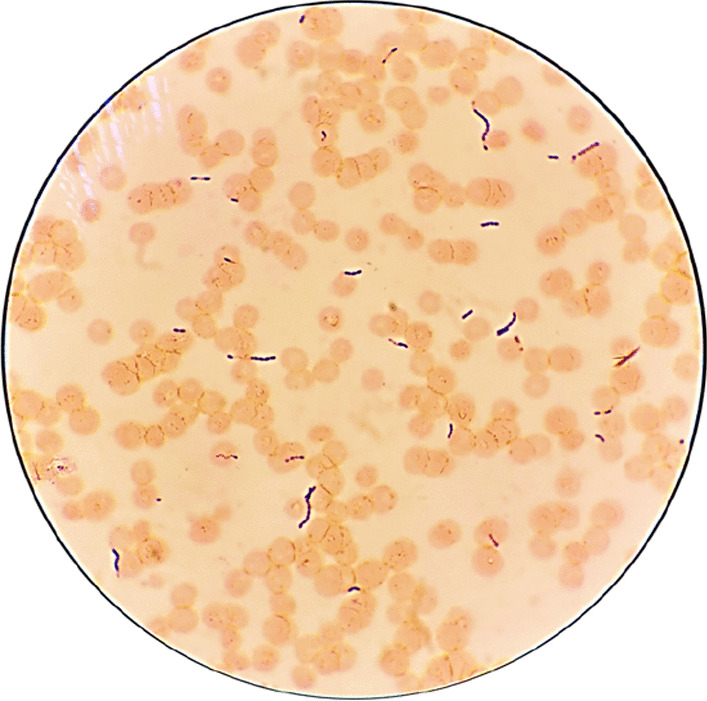
Gram stain from hemoculture showing gram-positive cocci in chain

After delivery, she received similar antibiotics. Cultivation of blood culture (taken at the time of fever), amniotic fluid obtained during Cesarean delivery, vaginal swab (obtained at the time of hospital admission) and placental swab (chorioamniotic membranes) revealed bacterial colonies identified as *S. mitis* (Fig. 1). Gram stain from enriched hemoculture showed Gram positive cocci in chains (Fig. 2). Interestingly, antibiotic sensitivity profiles from all specimens were similar (Table 1). Since her male neonate had respiratory distress, and severe hypoglycemia (blood sugar 16 mg/dL), which could be the sign of early onset neonatal sepsis, he was diagnosed with presumed sepsis requiring treatment. The diagnosis of presumed sepsis was based on intrapartum risk factor which involves the presence of intra-amniotic infection and the presence of respiratory distress as well as severe hypoglycemia requiring treatment in neonate [34]. Although, his WBC count, hemoculture and chest x-ray were within normal limits, infection could be present in the newborn. CRP value could be in normal limit due to low CRP value in preterm newborns. Therefore, he was given intravenous ampicillin and gentamicin for 7 days which is based on the recommendation by the American Academy of Pediatrics and the Centers for Disease Control and Prevention (CDC) [35, 36].

**Table 1:** Antibiotics sensitivity profile of the organisms derived from maternal blood, amniotic fluid, vaginal and placental cultures

Antibiotics	MIC	Interpretation
Penicillin	4 ug/mL	Resistant
Ampicillin	8 ug/mL	Resistant
Vancomycin	0.5 ug/mL	Sensitive
Clindamycin	> 2 ug/mL	Resistant
Daptomycin	≤ 0.5 ug/mL	Sensitive
Erythromycin	> 4 ug/mL	Resistant
Levofloxacin	> 8 ug/mL	Resistant
Tetracycline	> 16 ug/mL	Resistant

MIC: Minimum inhibitory concentration

**Fig. 3:**
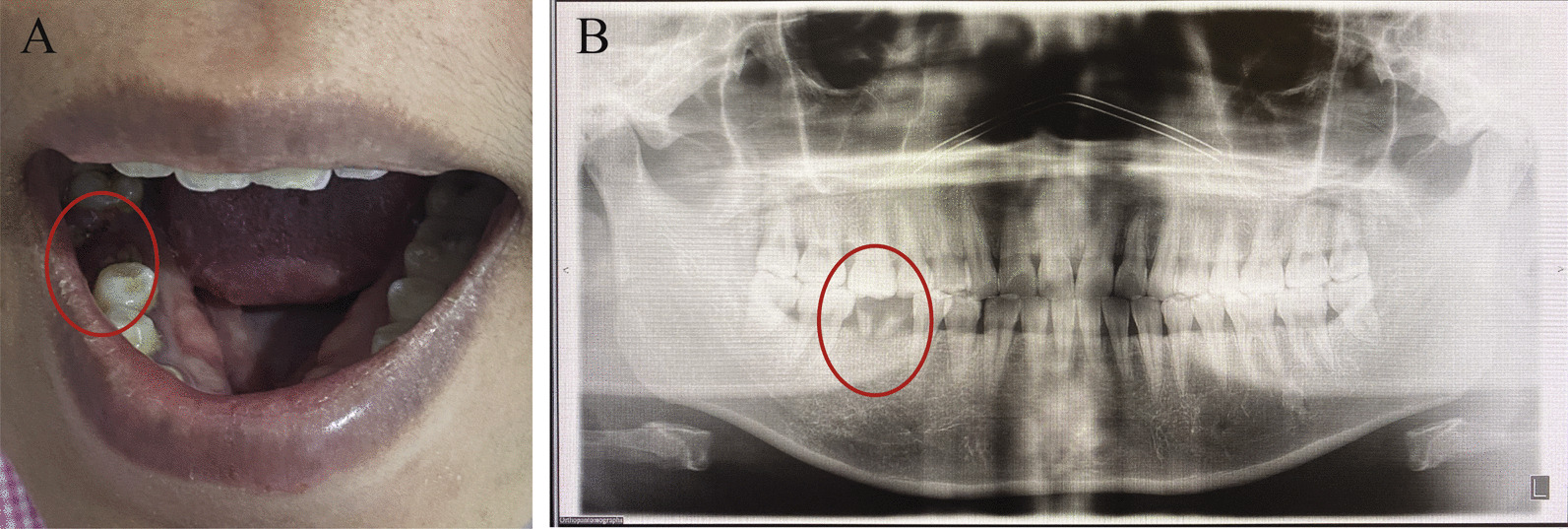
**A** Pulp necrosis with acute apical periodontitis, deep dental carries; **B** Dental X-ray: Fracture right lower molar tooth

**Fig. 4:**
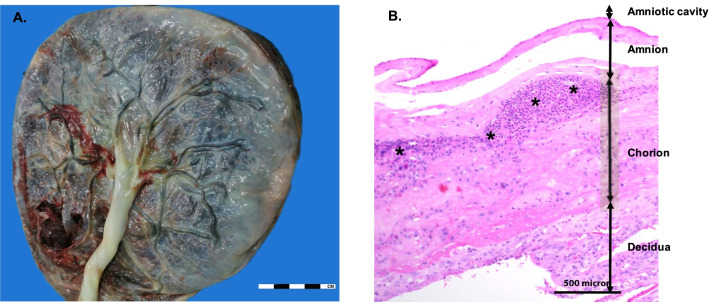
Pathology of chorioamnionitis: **A** Gross specimen of the placenta. Round shape of fresh placenta size 18.5 × 17 × 3 cm at fetal surface, shows turbid membranes. The tan umbilical cord inserts at central area; **B** Histopathology of chorioamniotic membranes. The membranes show acute chorioamnionitis stage 1 grade 2. Confluence of neutrophils infiltrates in the chorion or subchorionic space; * demonstrates neutrophils (H&E stain × 20). The Olympus BX53 microscope attached with Olympus DP73 digital camera and cellSens dimension software was used for microscopic study and photography

Since the patient was diagnosed with septicemia and *S. mitis* is usually found in the oral cavity, we consulted a dentist and an infectious consultant to determine the source of infection. Dental examination showed multiple dental caries with nearly exposed pulp, pulp necrosis and acute apical periodontitis (Fig. 3). Tooth extraction and curettage were performed. Vancomycin was intravenously administered for 1 week according to antibiotic sensitivity profile. At 6th week postpartum, her vaginal and dental caries bacterial culture demonstrated no *S. mitis,* and her clinical course was unremarkable. Placental histopathological examination confirmed acute histological chorioamnionitis stage 1 grade 2 (Fig. 4A and B). Gram positive cocci in chains was identified at chorioamniotic membranes. Retrospectively, the patient denied oral sexual intercourse during pregnancy. She reported that she subjectively had dental caries 1 year ago but did not get the treatment. The last time that she saw the dentist was more than 10 years. She did not receive dental treatment during this pregnancy. “Her partner denied having periodontal disease; however, he did not have a clinical assessment performed to determine periodontal disease status.”

## Discussion and conclusions

We reported a unique case with intra-amniotic infection, clinical chorioamnionitis and septicemia from *S. mitis* in a woman presenting with PPROM. Interestingly, the profiles of antibiotic sensitivity are identical among bacteria derived from amniotic fluid, vagina, chorioamniotic membranes and maternal blood, which suggests a similar bacterial strain. Multiple tooth decay and periodontitis were subsequently revealed. These observations suggested a link between periodontal disease and intra-amniotic infection. This is the first case report demonstrating maternal septicemia and intra-amniotic infection caused by *S. mitis* associated with periodontitis in the women presenting with PROM.

Periodontal disease is defined as a wide range of chronic inflammatory conditions of the gingiva (also called gums, the soft tissue surrounding the teeth), bone and ligament (the connective tissue collagen fibers anchoring the tooth to alveolar bone) that altogether support the teeth [37–39]. The initial stage usually begins with localized inflammation of the gingiva or “gingivitis” caused by bacteria in the dental plaque, a biofilm of microorganisms observed in the tooth surface [37–40]. Subsequently, untreated gingivitis leads to “chronic periodontitis” affecting surrounding epithelial tissues, the gingiva, alveolar bone and periodontal ligament, and eventually leads to tooth loss [37–39].

Accumulating evidence has shown that there is a relationship between periodontal disease and spontaneous preterm labor. The latest systematic review and meta-analysis including 16 case–control and 4 cohort studies demonstrated that periodontitis increases the risk of preterm birth about twofold (odd ratios 2.01; 95%; confidence interval 1.71–2.36) [41]. However, the definition of periodontitis is inconsistent among these studies [41]. Proposed mechanisms whereby periodontitis is associated with preterm labor are thought to be related to systemic inflammation [34, 39–41]. Oral pathogens could enter the maternal bloodstream and amniotic cavity causing inflammatory cascade in the fetoplacental unit triggering a common pathway of preterm labor [1, 2, 12, 42–44]. In animal studies, intravenous injection of *F. nucleatum* into pregnant mice resulted in premature delivery and stillbirths [42]. *F. nucleatum* colonizes in the uterus, amniotic fluid, and the placenta through hematogenous route [23]. In addition, oral pathogens such as *Streptoccoccus *spp*., F. nucleatum, Eikenella corodens, and Capnocytophaga* species are detected in the amniotic fluid of women with preterm labor or second trimester abortion [15–24]. In addition, dysbiosis of the placental microbiome has been reported to be associated with preterm birth [43–49]. Aagaard et al. characterized the placental microbiome using the metagenomic data derived from 16S rDNA as well as the whole genome sequencing and demonstrated that the placenta has a low abundance of microbiome composed of nonpathogenic commensal microbiota from the Proteobacteria, Tenericutes, Firmicutes, Bacteroidetes and Fusobacteria phyla. Importantly, such placental microbiome was shown to be most similar to the oral microbiome, and it is also associated with preterm birth [43]. Subsequently, several studies have determined placental microbiome dysbiosis in women with preterm birth [43–55]. However, it is still unclear whether periodontal therapy can reduce the frequency of preterm labor [46, 47].

*Streptococcus mitis* was first isolated and discovered by Andrews and Horder in 1986 from the human oropharynx [56]. The genus name ‘mitis’, based on the Latin root ‘mitis’, means “mild”, indicating low pathogenicity and virulence involved in different types of mild infections [26]. This species is a predominant pioneer colonizer of the oral cavity after birth and persists through life [57]. It also colonizes in other areas of the human body such as the skin, and the gastrointestinal and genital tracts as a part of the normal flora [26] although its presence in the vaginal flora is rare (2%) [58]. In the oropharynx, *S. mitis* is thought to form oral biofilms by supplying adherence sites for secondary colonizers [26, 59]. This bacterium can cause a variety of infectious complications including bacterial infective endocarditis, bacteremia, septicemia, meningitis, eye infections, and pneumonia, especially in elderly patients or immunocompromised patients [25, 28]. Currently, mortality rate from *S. mitis* bacteremia has not been reported. Nevertheless, mortality rates from viridans Streptococcus bacteremia range from 6 to 30% in immunocompromised hosts [60]. *S. mitis* colonizes the human oropharynx by several mechanisms including the expression of adhesins, production of immunoglobulin A proteases, and modulation of the host immune response [25, 26, 28]. Moreover, comparative genomic analysis confirms the presence of virulence genes such as hyaluronic acid synthesis-associated genes, which promote colonization and prevent bacteria from phagocytosis [61]. Importantly, bacteremia (including *S. mitis*) can occur after tooth brushing or other dental procedures [62–65]. The number of *S. mitis* significantly increases during pregnancy and is associated with dental caries, especially during the second and third trimester [66]. *S. mitis* has been infrequently isolated in the amniotic fluid of women with preterm labor (1.8%) [3, 5, 6], PPROM (2–3%) [4, 7] or short cervix (0.4%) [67].

Two previous reports discussed S. *mitis* in women with preterm labor and chorioamnionitis [27, 28]. Schmiedel et al. reported that *S. mitis* was identified from placental and fetal membranes by cultivation, fluorescence in situ hybridization and DNA sequencing techniques in women with clinical chorioamnionitis [27]. In this study, *S. mitis* could be identified only in the superficial layers of the fetal membranes but was found to be absent in the placental tissue, thereby indicative of ascending infection. However, there were no results of vaginal, blood or amniotic fluid microbiological examination. In addition, the source of *S. mitis* was not evaluated. A recent report by Hosseini et al. showed that *S. mitis* was isolated from amniotic membrane cultivation in a pregnant woman presenting with preterm labor at 21^+5^ weeks’ gestation who had no evidence of clinical chorioamnionitis [28]. The patient had a history of dental scaling and cunnilingus with her husband who had periodontal disease about 2 weeks prior to the onset of preterm labor. The authors hypothesize that acute histological chorioamnionitis caused by *S. mitis* is attributable to dental scaling or cunnilingus. However, this case was not confirmed to have periodontal disease or intra-amniotic infection since no amniotic fluid culture was performed. Even though one study demonstrated that *S. mitis* could be transmitted from a male partner to a female partner via vaginal intercourse, the mechanism has remained unclear [68]. *S. mitis* was also identified as one of the polymicrobial microorganisms detected in mid-trimester amniocentesis in asymptomatic pregnant women who subsequently experienced fetal death at 18 weeks’ gestation [29]. Other reports have shown evidence of intra-amniotic infection by viridans Streptococci and *S. mutans* in women presenting with preterm labor or short cervix [17, 18, 20, 22]. Interestingly, a successful eradication of such microorganisms is possible [17, 18, 22]. Table 2 described previous reports of *S. mitis* in acute chorioamnionitis cases.

**Table 2:** Reported cases of acute clinical chorioamnionitis caused by *Streptococcus mitis*

Author	Year	Country	Gestational age (weeks)	Clinical presentation	Suspected risk factors	Confirmed investigations	Treatment	Pregnancy outcome
Waites et al. [29]	1984	The United States	16	Intrauterine fetal death	Retained copper IUD	Positive culture from amniotic fluid No evidence of positive culture of *S. mitis* from vaginal swab, placenta, or fetus	Post-abortion antibiotics (Metronidazole + doxycycline)	Asymptomatic intrauterine infection with intrauterine fetal death
Schmiedel et al. [27]	2014	Germany	30	Sudden onset of maternal fever and fetal tachycardia	None	Positive culture from intraoperative swabs from placenta and fetal membranes with routine culture methods and visualized on FISH analysis Vaginal swabs with routine method was negative for *S. Mitis*	Cefuroxime	Acute chorioamnionitis was diagnosed and Cesarean delivery was performed at 30 weeks’ gestation
Hosseini, Hunt [28]	2020	Canada	21	Preterm labor (cervix dilated 4–5 cm) with afebrile with no evidence of any infectious symptoms	Recent dental scaling and recent cunnilingus with a partner known to have periodontal disease	*S. mitis* was isolated from a culture of amniotic membrane Placental pathology showed signs of acute chorioamnionitis including acute inflammation of placental plate chorion and acute funisitis		Delivered a male infant weight 510 g consistent with gestational age of approximately 22 weeks and died 1 h after delivery

FISH: fluorescence in situ hybridization

In conclusion, we reported a PPROM case with intra-amniotic infection and septicemia caused by *S. mitis*. This particular pathogen was systematically isolated from the samples of amniotic fluid, maternal blood, vaginal and chorioamniotic membranes with similar antibiotic sensitivity profiles indicating a similar strain. Subsequently, multiple dental caries and chronic periodontitis were identified and thought to be the possible source. Although the identification of *S. mitis* in dental plaque had not been performed in our case as we already initiated antibiotic therapy, it is possible that periodontitis found in the patient could have led to a bacteremia and septicemia resulting in intra-amniotic infection and PPROM. Altogether, these observations support the hypothesis that oral bacteria can gain access into the amniotic cavity through hematogenous dissemination [43, 49]. Therefore, prenatal care should include oral cavity examination to ensure good dental hygiene [69–72]. If possible, dental care should be performed prior to pregnancy to prevent maternal septicemia or other associated infections caused by oral bacteria and to maintain overall health of pregnant woman.

We reported a case with intra-amniotic infection, clinical chorioamnionitis and septicemia caused by *S. mitis*. Dental caries or chronic periodontitis may be a source of intra-amniotic infection, thereby highlighting an association of periodontal disease and preterm labor/PROM syndrome. This is the first case report demonstrating *S. mitis* septicemia and clinical chorioamnionitis that develops from chronic periodontitis and tooth decay. We, here, support proper dental hygiene [72] and promote dental care, including identification, prevention and treatment of oral diseases, before and during pregnancy.

## Data Availability

All data generated or analyzed during this study are included in this published article [and the additional information files].

## Abbreviations

CRP: C-reactive protein
PROM: Prelabor rupture of membranes
PPROM: Preterm prelabor rupture of membranes
WBC: White blood cell
